# Urinary Tract Infections: Etiology and Emerging Therapeutic Approaches

**DOI:** 10.3390/antibiotics15070686

**Published:** 2026-07-13

**Authors:** Rivka F. Kayser, Danielle Kamsan, Zvi G. Loewy

**Affiliations:** 1 Touro College of Pharmacy, Touro University, New York, NY 10036, USA; 2 School of Medicine, New York Medical College, Valhalla, NY 10595, USA

**Keywords:** antibiotic resistance, biofilm, dysbiosis, microbiome, quorum sensing

## Abstract

Urinary tract infections (UTIs) are considered a significant public health issue due to their high incidence and prevalence, most notably among women. The potential for complications, including recurrent infections and antibiotic resistance, negatively impacts patient quality of life and correspondingly results in elevated healthcare costs. Efficacy of antibiotic pharmacotherapy has been shown to be limited in the treatment of UTIs. The use of antibiotics has been further exacerbated by the unprecedented spread of antimicrobial-resistant organisms. This paper reviews the microbiome specific to UTIs and the formation of biofilms in UTI patients, and presents the current status of novel biological and chemical approaches designed to inhibit and eradicate biofilms in UTI patients.

## 1. Introduction

Urinary tract infections (UTIs) are exceedingly common bacterial infections [1]. The worldwide annual incidence of UTIs is greater than 150 million cases [2]. UTIs are heterogeneous with respect to their severity, ranging from simple (urethritis and cystitis) to severe (bacteremia and septic shock) [1]. Based on United States epidemiological data, one in three women have a minimum of one UTI that requires antibiotic treatment [3]. UTIs, however, present a significant issue in pharmacotherapy attributed to antibiotic resistance, resulting in treatment failure and patient morbidity [4]. Importantly, UTIs result in an annual medical treatment cost exceeding 4.8 billion dollars in the United States [5].

Discovery and application of antibiotics are among the most significant advancements in modern medicine. Antibiotics have greatly reduced morbidity and mortality from bacterial infections. Importantly, the use of antibiotics is justified only when truly needed; unnecessary uses result in antibiotic resistance [6]. Resistant organisms can spread via direct contact, healthcare environments, or community transmission.

Antibiotic resistance is the capability of bacteria to survive and proliferate in the presence of antibiotics that would normally inhibit their growth or eliminate them. Resistance develops through various mechanisms, including genetic mutations, production of enzymes that degrade antibiotics, efflux pumps, horizontal gene transfer, and adaptive physiological changes. Resistance is typically identified through in vitro susceptibility testing. Because of antibiotic resistance, extended illness and increased mortality worldwide ensue [7].

Results from a 2016 national study estimated that approximately 30% of outpatient antibiotic prescriptions were unnecessary [8]. As of 2022, Centers for Disease Control and Prevention (CDC) surveillance data show that nearly 28% of outpatient antibiotic prescriptions are unwarranted. Significantly, 29.5% of these prescriptions involved broad-spectrum antibiotics, which are associated with a high risk of promoting resistance [9].

Globally, antibiotic resistance is increasing rapidly. In 2021, bacterial antimicrobial resistance was linked to an estimated 4.71 million deaths, including 1.14 million directly caused by resistant infections [10]. Adults older than 70 years of age have had more than an 80% increase in deaths attributed to AMR during the same timeframe [11].

There is elevated concern about increasing resistance in Gram-negative pathogens and a paucity of new antibiotics in development [11]. Antibiotic use has also grown significantly, resulting in a 16.3% increase from 2016 to 2023 [12]. Antibiotic use manifests selective pressures that cause bacteria to develop survival strategies, making resistance a public health issue. As long as antibiotics are used, bacteria will continue to evolve and develop resistance strategies [13].

The impact of antibiotic resistance in UTIs is significant. In this paper, we aim to describe the composition of urinary biofilms implicated in UTIs and present novel approaches that are being explored, evaluated, and developed to curtail the incidence of UTIs. Clearly, viable alternative antimicrobials are needed.

## 2. Methods

Two general questions guided the review: 1. what are the characteristics of the microbiome implicated in UTIs, and 2. what are the emerging therapeutic approaches for UTIs? For the searches, the following parameters were used: 1. microbiome articles; and 2. interventions.

### 2.1. Eligibility Criteria

The searches were restricted to articles published in English with a date range of 2014–2026. Opinion articles, letters, and conference abstracts were excluded due to the risk of bias associated with those articles. Studies on the use of smoke of cannabis plants were also excluded.

### 2.2. Information Sources and Search

The searches in the literature databases were performed by three independent investigators. The search was carried out using PUBMED, MEDLINE, and EMBASE. The search strategy was based on MeSH terms of PubMed, using the search terms presented in Table 1. The reviewers also hand-searched the reference lists of the included articles to identify additional papers.

### 2.3. Selection of Sources of Evidence

**Figure 1:**
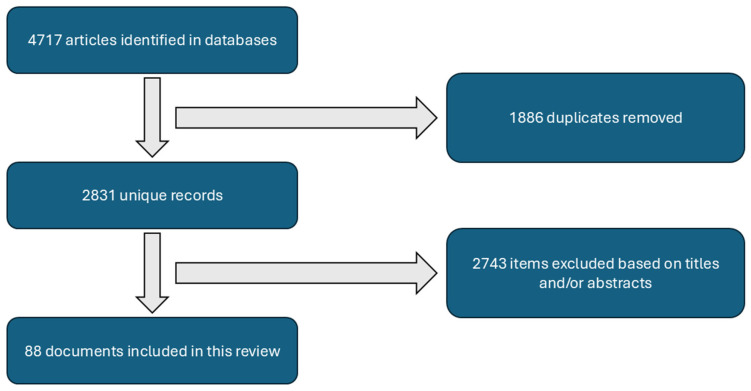
Search results.

Retrieved articles were classified as included, excluded, or uncertain. Full-text versions of the included and uncertain articles were selected for further eligibility screening. A total of 2831 unique articles were identified, with 2743 articles excluded based on their titles and/or abstracts (Figure 1).

## 3. Results

### 3.1. Biofilm Life Cycle

**Figure 2:**
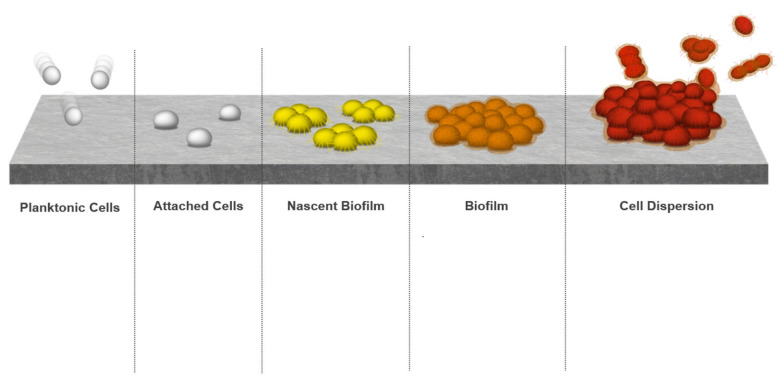
Stages of biofilm formation.

Biofilms are communities of bacteria that begin forming when planktonic bacteria attach onto either a natural tissue or a synthetic substrate and begin replicating rapidly. In a nascent biofilm, bacteria begin to engage in a complex communication system known as quorum sensing (QS). Chemicals, called autoinducers (AIs) are secreted by the bacteria and sensed by each other. The increased concentration of these autoinducers allows the bacteria to sense the increased density of the biofilm. This triggers certain processes that work only when bacteria are found in a more densely populated aggregation. These processes enable the survival and growth of the biofilm. One of these processes is the secretion of an extracellular matrix comprising polysaccharides, proteins, nucleic acids, and lipids, which encapsulates, fortifies, and nourishes the biofilm [14]. The biofilm grows into a robust, tight complex of microorganisms that can withstand the body’s defenses and is resistant to conventional antibiotics. At the conclusion of a biofilm life cycle, the mature biofilm releases bacteria, which then migrate to other sites to begin the process anew (Figure 2).

### 3.2. Urinary Biofilms

In the human body, biofilms are prevalent in the oral cavity, ear, skin, respiratory tract, gastric system, and urogenital system. Urinary tract infections are a common infection, especially in females. When biofilms develop, infections are no longer simple to treat. There can be higher rates of UTI recurrence and increased incidence of antimicrobial resistance. When a person has a catheter in place, it is even more susceptible to biofilm development, and this is a major cause of catheter-associated UTIs (CAUTIs).

Several different bacteria are implicated in urinary tract infections and biofilms, including uropathogenic *Escherichia coli*, *Klebsiella pneumoniae*, *Proteus mirabilis*, *Enterococcus faecalis*, *Staphylococcus epidermidis*, *Staphylococcus saprophyticus*, and *Staphylococcus aureus* [15]. These bacteria are equipped with virulence factors that allow them to cause infection and sometimes even initiate a biofilm. For example, uropathogenic *E. coli*, the most common bacteria responsible for localized UTIs and systemic UTIs, has adhesins that are key to enabling it to attach to surfaces and begin the biofilm formation process. Its flagellar motility allows it to move and initiate new biofilms. *Klebsiella pneumoniae*, with its pili and thick polysaccharide capsule, is known for its strong adhesiveness to catheters and medical devices and is prone to forming biofilms on these surfaces. These bacteria, when incorporated in a biofilm, are significantly more probable to display multidrug resistance [16]. *Candida albicans* is a common resident of skin and mucous membranes. It is also an opportunistic fungus that causes candidiasis in different tissues, including the oral cavity, genitourinary tract, ears, bones, eyes, wounds, and blood [17,18]. *C. albicans* is the primary cause of fungal urinary tract infections and accounts for up to 25% of all CAUTIs and 10–20% of all nosocomial infections, with a significant risk of development into a systemic infection with high mortality rates [19].

Many reasons have been proposed as to why urinary catheters make patients susceptible to urinary tract infections, including the “absence” of the natural sphincter barrier allowing microbes to migrate upwards and cause infection [20]. Additionally, in healthy patients without a catheter in place, upon the arrival of microbes at the bladder, the body triggers an inflammatory response, recruiting neutrophils for the removal of pathogenic bacteria. When a foreign object is implanted, the body cannot recognize that there is a pathogen trying to attach, and it will not activate this immune response, allowing the bacteria to generate biofilms. These biofilms can lead to UTIs that are especially difficult to treat. Biofilms manifest several sophisticated mechanisms for evading antibiotics [21].

### 3.3. Eubiosis and Dysbiosis

**Figure 3:**
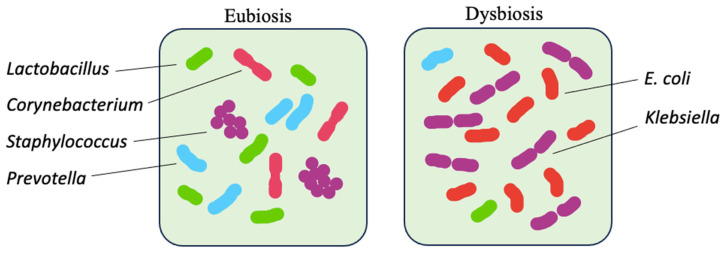
Contrasting eubiosis to dysbiosis.

The natural urinary tract microbiome plays a crucial role in urinary health by preserving a functional environment that can prevent the proliferation of pathogens. Eubiosis is a term used to describe the natural microbiological state of an environment in the body. Normally, the urinary microbiome comprises a diverse, well-balanced population of bacteria. Although each person has a unique urinary microbiome, within each person’s unique urinary microbiome composition there are certain core bacteria, such as *Lactobacillus*, *Corynebacterium*, *Staphylococcus*, *Streptococcus*, *Veillonella*, and *Prevotella*, which are normally resident in all humans, except for some sex-specific differences (Figure 3). *Lactobacillus* is particularly abundant in females, while *Corynebacterium* or *Streptococcus* are predominately found in males. Dysbiosis manifests when the balance is disrupted, resulting in an overabundance of uropathogenic bacteria and a less diverse population [22]. Dysbiosis of the urinary tract can lead to urinary tract infections; bacteria such as *Escherichia coli*, *Klebsiella pneumoniae*, *Proteus mirabilis*, and *Enterococcus faecalis* have been identified in urinary tract infections.

The risk of urinary tract disorders can frequently be assessed by analysis of the composition of the urine microbiome. When the native microbiome is altered, dysbiosis manifests. The relative abundance of certain bacteria can predict future risk of bladder dysbiosis. Women with a lower abundance of *Lactobacillus*, notably *Lactobacillus crispatus*, determined by 16S rRNA gene sequencing and quantitative urine culture techniques, were found to have an increased risk of bladder disorders, including urinary incontinence [23]. A disruption in the diversity of the urine microbiome has been implicated in the pathogenesis of rUTIs [21]. This is the result of several mechanisms, including migration of resistant uropathogenic bacteria from the gut or reinfection from an external source [23,24]. Microbial profiling of several human microbiomes has shown significant commonalities among the urine, gut, and vaginal microbiomes. Using the 16S rRNA gene sequencing method, 64.1% of bacteria in the urine were found to be conserved with the human gut microbiome [25]. This suggested that the origin of the urine microbiome was found to be mostly of gastrointestinal origin [23].

### 3.4. Urinary Catheters and Biofilm Growth

CAUTIs are the most common healthcare-associated infections, and account for 1 million cases per year in the United States [26]. They are the most common cause of secondary bloodstream infections. In total, 3–10% of residents in long-term care facilities are managed with chronic indwelling catheters [27,28]. The associated costs of preventable CAUTIs are estimated to range from $115 million to $1.82 billion annually [29]. Risk factors for CAUTIs include age, female gender, diabetes, and prolonged catheterization time [30]. The duration of catheterization is the most important factor in the development of bacteriuria, with a risk of 3–7% daily [28]. A mean of 3.2 urinary tract infections per 1000 catheter days was reported in long-term care facilities in a US study [31]. In the intensive care unit (ICU), where infection rates are 3–5 times higher than other hospital patient care areas, the incidence of CAUTIs is 7.78 per 1000 catheter days [32]. CAUTIs in ICUs are associated with prolonged lengths of stay, elevated healthcare expenditures, and the overuse of antibiotics [33,34].

UTIs generally occur through urethral exposure to rectal flora, followed by microbial migration to the bladder, adhesion, and colonization [35]. Invasion of the bladder is then facilitated by pili and adhesins, and the beginning of neutrophil infiltration. Bacteria proliferate and form biofilms; bacterial proteases and toxins initiate epithelial damage. Regardless of the presence or absence of a urinary catheter, the fundamental steps of infection are the same. Catheters serve as a breeding ground for infection, increasing the risk for a UTI. In addition, catheters may also irritate the uroepithelium, thereby disrupting the physiologic mucopolysaccharide coating, and making it susceptible to bacterial adhesion and entry [35,36]. The strong immune response to catheterization results in fibrinogen accumulation on the catheter, providing an environment for adherence by uropathogens. Although *Enterococcus faecalis* is unable to grow in urine or bind to catheter material in vitro, citrate and aspartate are significant for growth of *E. faecalis* in human urine, and manganese appears to be a limiting factor, as it grows in fibrinogen-supplemented urine and can adhere to a fibrinogen-coated catheter [37]. Adherence is a key initial step in urinary tract infection [15]. In the presence of a urinary catheter, UTIs may be initiated upon bacterial adherence to the catheter, with subsequent biofilm formation [38].

Microbial species utilize varied mechanisms for biofilm formation. Uropathogenic *Escherichia coli* (UPEC), the most common causative organism for CAUTIs, utilize pili, antigen 43, and curli to promote interbacterial attachment, bacterial-surface adhesion, and subsequent biofilm formation [15]. Specifically, UPEC catheter biofilm formation is dependent on type 1 pili UPEC biofilm formation, and is regulated by oxidative stress, iron sensing, and quorum sensing [39,40,41]. *Pseudomonas aeruginosa* manifests biofilm formation on urinary catheters through a number of mechanisms, including alginate production, quorum sensing, and surface hydrophobicity modulation [41]. Biofilms contribute to the pathogenicity of *P. aeruginosa* and lead to persistent or recurrent infections. *Proteus mirabilis* is a urease producing species. Hydrolysis of urea increases urinary pH, resulting in the formation of calcium crystals and magnesium ammonium phosphate precipitates. This results in the formation of crystalline biofilms on catheters [15].

### 3.5. The Human Microbiome in the Treatment of Urinary Tract Infections: Case Studies

**Figure 4:**
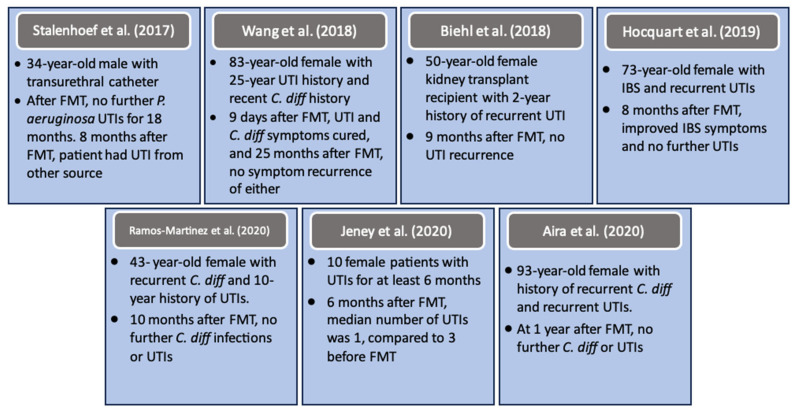
Summary of fecal microbiota transfer case studies [43,44,45,46,47,48,49].

Theoretically, since dysbiosis of the urinary tract leads to urinary tract infection development, replenishing the natural bacteria at that site should therefore restore the body’s health and its ability to fight the infection. Interestingly, beyond that, there appears to be a strong connection between gut microbiota and urinary microbiota. A healthy, robust microbiome at one site may manifest in the other, and the inverse may be true as well. Researchers have wondered if implanting feces with healthy microbiota into a patient prone to urinary tract infections will cause the patient to develop a healthier urinary microbiome, and if the patient will experience a decline in subsequent urinary tract infections. This procedure, known as Fecal Microbiota Transplantation (FMT), has been utilized in different forms throughout the centuries, and eventually gained traction after it was approved by the FDA in 2013 for the treatment of recurrent and refractory *Clostridium difficile* infections [42]. As reported in the following preliminary case studies, FMT may also have a benefit in patients experiencing recurrent UTIs, not just in those suffering from *C. difficile* infections (Figure 4).

A study by Stalenhoef et al. [43] presented a case report regarding a 34-year-old male with Type 1 Diabetes Mellitus who was on peritoneal dialysis. The patient developed a febrile urinary tract infection shortly after a transurethral catheter was placed due to neurogenic bladder dysfunction. The patient was treated empirically with ceftazidime. Urinary cultures, as well as rectal swabs and the catheter exit site, were positive for blaVIM carbapenemase-producing *P. aeruginosa*, which is resistant to many antibiotics. The patient was treated with IV colistin for two weeks. The same infection recurred two times after that, and the patient was treated similarly. After the third infection, the treatment was altered. It was thought that this was a case of reinfection from gut bacteria. The patient was therefore scheduled for FMT. Six weeks after the last colistin course, the fecal microbiota transfer was infused. The fecal donor was an unrelated healthy volunteer. The recipient underwent a full colon lavage, after which 300 mL of fecal suspension was infused in his duodenum through a nasoduodenal tube. Stool samples were collected from the patient prior to infusion, and after 1 week, 2 weeks, 1 month, 2 months, and 3 months, and the samples were screened for the presence of multidrug-resistant bacteria. The only adverse event the patient experienced was loose stools for three days. Stool culture before FMT was negative for the strain of MDR *P. aeruginosa* and remained negative for the duration of follow-up (3 months). No infections due to *P. aeruginosa* were noted for the next 18 months.

Wang et al. presented an 83-year-old female patient with a 25-year history of recurrent UTIs [44]. Given the patient’s allergies to ciprofloxacin, nitrofurantoin, and sulfa drugs, treatment options for UTIs were severely limited. UTIs were frequent, and urine cultures showed MDR *E. coli* on several occasions. As time progressed, there were several other bacteria isolated with increased levels of drug resistance; UTIs occurred more frequently. For the 2 years prior to the study intervention, the patient was on continuous antibiotic treatment. The medical leads on the case explored many methods to prevent the UTIs from recurring, including solifenacin succinate, vaginal estrogen, increased fluid intake, scheduled voiding, stool softeners, methenamine hippurate, and vitamin C, but these methods were to no avail. The patient developed several *C. difficile* infections and was treated initially with metronidazole and subsequently with oral vancomycin. The patient continued to have multiple UTIs, with decreasing intervals between episodes. After a third *C. difficile* relapse, the patient was scheduled to receive a fecal microbiota transplantation. Stool from an unrelated donor was administered to the patient via colonoscopy. By 9 days after the FMT, the patient was cured of all *C. difficile* and UTI symptoms, and at 25 months after the procedure, there were no recurrences. For this patient, FMT appeared to be effective in reducing UTI recurrence.

Biehl described a case study of a 50-year-old female kidney transplant recipient on immunosuppressive therapy [45]. The patient was experiencing recurrent UTIs for 2 years, with the last few episodes showing presence of ESBL *E. coli.* Prophylactic antibiotic treatment and cranberry concentrates did not prevent episodes; the patient was scheduled for FMT based on previous case studies showing FMT as a possible method for preventing recurrence of UTIs. Both prior to and following the FMT procedure, midstream urine samples, stool samples, and vaginal swabs were taken. These samples were used to evaluate whether there was a successful transfer of the donor’s microbiota to the recipient. When the patient presented for FMT, she had been asymptomatic for 2 weeks. Patient received FMT from a healthy donor via oral administration of frozen capsulized microbiota on two consecutive days. Nine months after the procedure, the patient had no occurrence of UTI symptoms and did not receive antibiotics, highlighting the successful transfer of the donor’s microbiota.

Hocquart described a case study of a 73-year-old woman with a past medical history that included severe irritable bowel syndrome (IBS) and recurrent UTIs [46]. Because the symptoms of IBS were not ceasing, it was decided that FMT would be administered. The patient received FMT with 400 mL of fecal infusion from a healthy donor via a nasogastric tube after a bowel lavage. The patient’s stool was collected prior to and following the transplantation for analysis. Eight months after FMT, many favorable outcomes were noted. The patient was having less stools per day (3–4 compared with 10–15 pre-transplantation), and the stool consistency was improving. The intestinal incontinence that the patient previously experienced decreased and correspondingly required fewer antidiarrheal medications. The patient had no more UTI episodes from the time of transplantation to the time of monitoring after 8 months, and there was no bacterial growth noted in urinalysis. The patient’s quality of life also improved significantly. In terms of the stool analysis, there were 12 bacteria species identified in the infusion sample that also appeared in the post-transplantation sample. The 12 identified bacteria species were: *Anaerococcus vaginalis*, *Bifidobacterium longum*, *Campylobacter ureolyticus*, *Intestinimonas massiliensis*, *Lactobacillus pentosus*, *Oscillibacter massiliensis*, *Phascolarctobacterium faecium*, *Prevotella denticola*, *Provencibacter massiliensis*, *Streptococcus agalactiae*, *Streptococcus constellatus*, and *Streptococcus parasanguinis.* These 12 species had not been present prior to transplantation, suggesting successful transfer of these bacteria from the donor, and were potentially being responsible for the improved outcomes.

Ramos-Martinez described a case regarding a 43-year-old patient with a history of recurrent *C. difficile* infections and a ten-year history of recurrent UTIs [47]. The urinary tract infections were caused by different bacteria over the years. The patient had a long-term suprapubic catheter in place, which may have contributed to the development of the recurrent urinary tract infections. After the third episode of recurring C. difficile, the patient was scheduled to receive a fecal microbiota transfer. Patient was given fresh stool provided by his wife, which was administered via colonoscopy. The procedure successfully prevented the recurrence of *C. difficile;* 10 months after the procedure, the patient also had not experienced any more UTIs despite the long-term suprapubic catheter.

Jeney reported a prospective case series of 11 female patients (10 of whom completed the study in its entirety) with a median age of 70 [48]. These women had been experiencing recurrent urinary tract infections while on antibiotics for at least 6 months. The investigators wanted to determine if fecal microbiota transplantation could reduce the incidence of UTIs by restoring the normal gut microbiome. The primary outcome of the study was the number of symptomatic UTIs that were proven by culture and treated with antibiotics for a time span of 6 months before transplantation, compared to 6 months after transplantation. The median number of UTIs per participant before transplantation was three, compared to only one after transplantation. Before transplantation, six participants had at least one urine culture containing extended spectrum beta-lactamase (ESBL)-producing bacteria. At 3 months post transplantation, only one patient had ESBL-producing bacteria cultured, and at 6 months post transplantation, two patients did. There were no significant adverse events noted due to FMT. The study provided a strong rationale for further research into this potential treatment modality.

A study by Aira focused on a 93-year-old female patient with a history of end sigmoid colostomy for acute diverticulitis, recurrent UTIs, and recurrent *C. difficile* infections treated with ciprofloxacin [49]. Traditional antibiotic treatments did not work to prevent the recurrence of *C. difficile* in this patient; as such, the patient received fecal microbiota transferences (FMTs) via colonoscopy through a colostomy. In the year after FMTs, the patient experienced no further recurrences of *C. difficile* infections. Interestingly, the patient also did not experience any more urinary tract infections. In healthy humans, the GI tract is predominately populated with Bacteroidetes and Firmicutes. In this patient’s fecal sample, before FMTs were performed, there was marked dysbiosis, with Enterobacteriaceae, specifically *Klebsiella*, as the most predominant phyla. The samples collected after FMTs showed a significant decrease in the abundance of Enterobacteriaceae, and an increase in overall healthy microbial diversity. The fact that FMT also appeared to prevent UTI recurrence is suggestive that there is a link between gut microbial health and the microbial health of the urinary tract.

### 3.6. Rare Etiologies of UTI

Sometimes, a UTI can be difficult to treat because it was caused by a bacterial species that is not commonly implicated in UTIs, and therefore standard antibiotic treatment will not work. Mazumder et al. [50] reported that a 63-year-old female presented to the clinic in January 2022 with symptoms such as dysuria, mild lower abdominal pain, fever with chills, and lethargy. Having classic symptoms of a UTI, the patient was treated empirically with intravenous ceftriaxone for seven days. This treatment did not work, and the symptoms persisted. The patient was admitted to the hospital, and a clean catch midstream urine sample was cultured. The causative organism was found to be *Klebsiella aerogenes*, a member of the Enterobacteriaceae family. The bacteria was resistant to most drugs tested and was only found to be susceptible to carbapenems and polymyxins. Based on the susceptibility data, the patient was given meropenem intravenously for 14 days. The patient was successfully treated and did not experience recurrences throughout follow-up.

A case report by Barrios-Villa et al. [51] showed another instance where bacteria not typically causative of UTIs were isolated. A married 40-year-old female patient with renal artery stenosis was experiencing UTI symptoms on a frequent basis (at least 5 times a year) since having a catheter inserted 10 years prior. Her symptoms were mainly dysuria and low volume micturition. She had repeatedly experienced negative urine cultures despite her symptoms. Intracellular bacterial communities were found in the bladder urothelium and had to be released in order to show a positive bacterial culture. Through various complex microbiological identification methods, including Gram staining, the species responsible for this urinary tract infection were identified as *S. aureus*, *S. epidermidis*, *S. simulans*, and *Streptococcus agalactiae*. These showed resistance to some antibiotics; however, they demonstrated susceptibility to nitrofurantoin and several other antibiotics—the patient was undergoing treatment with nitrofurantoin at the time the case report was written. These bacteria are not commonly found in urinary tract infections, and this underscores the importance of “thinking outside the box” when treating recurrent UTIs.

### 3.7. Emerging Preventative Treatments

Due to the growing concern of antibiotic resistance, particularly multidrug resistance, Sihra et al. [52] explored potential preventative options. Some of these are completely natural methods and behaviors, and proper patient counseling could improve outcomes without resorting to antibiotic use. Avoiding certain practices that have a known link to UTIs, such as spermicide-based contraception use, can prevent the development of UTIs and therefore prevent the need to use antibiotics that contribute to the drug-resistance problem. Certain dietary measures may help prevent UTIs, although these have not been proven yet. These include proper hydration, which was only seen effective based on small, poorly performed studies, and vitamin C intake, which has also not been thoroughly studied and cannot be labeled as effective yet. Another well-known method for prevention of UTIs is the intake of cranberry-based products. A component in cranberries, proanthocyanidin, is thought to impair the adhesion of certain uropathogenic bacteria to the urinary epithelium. As such, 240 mL of cranberry juice cocktail is thought to be sufficient to exert this anti-adhesive effect. However, although smaller studies have shown cranberries to be effective in reducing UTIs, randomized controlled trials have had mixed results, and there were limitations associated with these trials; therefore, the data is not clear in either direction. However, there were few adverse events associated with cranberry use; therefore, it can be mentioned to patients as a potential option, with the caveat that it has not been sufficiently proven in well-powered trials.

Another method of UTI prevention discussed was probiotics (both oral and vaginal), which did not show improvement in UTI reduction; perhaps a large-scale study would address some of the limitations in these studies and show different results. D-mannose has shown promise in several small in vivo trials [53]. Its mechanism of action is thought to be mainly the inhibition of bacterial adhesion to urothelial cells, and it appears to be effective in reducing the incidence of UTI recurrence. Methenamine hippurate, topical estrogen, and intravesical glycosaminoglycans (hyaluronic acid plus chondroitin sulfate) are other options that have been studied preliminarily, but these also require larger randomized studies to confirm the benefit of these agents. The common theme between these alternative methods of UTI prevention is that they have either shown contradictory results in different trials or have not been well-powered to detect a statistically significant result. If future studies show some of these methods to truly reduce UTI occurrence, this could be a major victory in the fight against antimicrobial resistance.

### 3.8. Microbial Treatment

In addition to the connection between gut microbiota and urinary health, as demonstrated in the case reports of fecal microbiota transplantation, the vaginal microbiome also contributes a vital role in urinary health. In a phase 2 randomized control trial, Stapleton et al. demonstrated that vaginal *Lactobacillus* repletion after a UTI could prevent the recurrence of UTIs in the future [54].

A total of 100 young female patients, who were experiencing at least their second UTI in a year (note: recurrent UTI is defined as two or more UTIs within a 6-month period, or three or more UTIs in a year), were enrolled in the study. At first visit, patients were treated for their current UTI according to standard of care. Then, 7–10 days after treatment, patients returned, and were randomized 1:1 to receive either Lactin-V, which contains a strain of *Lactobacillus crispatus* from a healthy female vagina, or a placebo. Lactin-V or placebo were self-administered as an intravaginal suppository once a day for 5 days. Then, it was taken once a week for ten weeks. After one week, as well as at 10 weeks, patients returned for follow-up.

In the Lactin-V group, seven patients (15%) had at least one UTI compared to 13 patients (27%) in the placebo group. In the Lactin-V group, 41 out of 48 patients displayed high levels of *L. crispatus* colonization in the vagina compared to 32 in the placebo group. The patients who received Lactin-V and displayed high levels of *L. crispatus* colonization vaginally during follow-up experienced a significant reduction in UTIs.

Although this was a very small-scale study, the preliminary results can provide the impetus for larger and longer studies. Microbiota restoration could be a very useful treatment modality if further proven to be effective, especially when noting the efforts and resources being dedicated to reducing antimicrobial resistance.

### 3.9. Bacteriophage Therapy

Since their initial discovery in the early 20th century, phages have been explored as remedies for many infections, including UTIs. Several characteristics of phages make them potential therapeutic candidates, including their specificity, safety, ubiquity, and self-dosing (replicating) capabilities.

Biofilms in CAUTIs are notoriously difficult to treat with antibiotics, often requiring removal of the catheter for treatment [55]. The extracellular matrix produced by the biofilm prevents penetration of some antibiotics [13,14,15]. Some bacteriophages possess factors, including cell wall degrading polysaccharide depolymerases, that enable bacterial killing within biofilms [56,57,58]. Eradication of biofilms by phages has been demonstrated in vitro, with some phages having the capacity to both disrupt established biofilms as well as inhibit biofilm formation on catheters [58,59,60].

Initial investigations with phages demonstrated examples of positive outcomes; however, the use of bacteriophages as therapeutics rapidly declined following the large-scale introduction of antibiotics in the 1940s. Contributing to the decline of bacteriophage use was the observation that bacteria can rapidly develop resistance to phage under selection pressure.

Several case reports on the use of bacteriophages for urinary infections have been published in recent years, some of which were successful while others resulted in failure [61]. Many of these case studies demonstrated how phage therapy can be complicated by the development of phage resistance [62]. Resistance mechanisms include gene mutations, clustered regularly interspersed short palindromic repeats (CRISPRs), and restriction–modification.

In a case study reported by Leitner et al., the investigators aimed to determine whether bacteriophages could be effective in eradicating urinary tract infections in humans in a randomized, placebo-controlled double-blind trial [63]. Adult males who were scheduled to get a transurethral resection of the prostate (TURP) and had developed cystitis before receiving the procedure were enrolled in this study. This patient population was chosen because they are typically given a suprapubic catheter for low pressure irrigation, and this would enable them to receive therapy via intravesical instillation. The patients were enrolled 1:1:1 to receive 20 mL of either a Pyo bacteriophage cocktail (pyophage), a placebo of bacteriophage buffer, or systemically given antibiotic treatment as standard of care via the systemic route. The pyophage cocktail was filled with individual bacteriophages that covered species, such as *Enterococcus* spp., *Escherichia coli*, *Proteus mirabilis*, *Pseudomonas aeruginosa*, *Staphylococcus* spp., and *Streptococcus* spp. Only patients who had UTIs caused by these pathogens were included in the study. For the pyophage and placebo groups, intravesical instillation was performed twice a day for seven days, starting from the day after TURP surgery. In total, 28 patients were given the pyophage cocktail, 32 were given the placebo, and 37 were given antibiotics. At day 7 of treatment, normalization of urine culture, as well as symptom relief, was experienced by 18% of patients in the pyophage group, 28% of patients in the placebo group, and 35% of patients in the antibiotics group. The difference between these groups was not considered significant. Bacteriophage therapy was considered non-inferior to standard antibiotic treatment, but it was not considered superior to placebo [54]. The similar results observed between bacteriophage and placebo may be due to the therapeutic effect of simply irrigating the bladder. Although in this case, the study did not prove the efficacy of bacteriophage therapy; it is a concept that may be proven effective in larger, more well-powered clinical studies.

### 3.10. Antimicrobial Peptides

Antimicrobial peptides (AMPs) are small, positively charged, naturally occurring chains of amino acids that form peptides and are responsible for maintaining a healthy biome. They are known as the human body’s “endogenous antibiotic system” due to their broad-spectrum bactericidal effects [64]. Their ability to disrupt a bacterial cell’s membrane by forming pores and making it unstable, resulting in cell death, make AMPs a viable approach to overcome bacterial resistance. AMPs can be manipulated, amplified, or synthesized to provide unconventional therapies to combat antimicrobial resistance. Amplification of AMP activity has been studied with histone deacetylase inhibitors (HDACis) to evaluate if they can increase antimicrobial activity by upregulating RNase 4 and 7, the enzymes responsible for amino acid and peptide production specifically in the urinary tract [56]. By introducing HDACis, histone acetylation activity increased, resulting in the promotion of RNase 4 and 7 transcription. This resulted in a quantitative boost of AMP production in the urinary tract, exhibiting antimicrobial activity specifically toward recurrent UTIs [65]. These observations showed that AMP amplification could be an alternative to traditional antibiotic therapies and thus mitigate antibiotic resistance.

Additional AMPs studied include Human Neutrophil Peptides 1–3 (HNP1–3). These AMPs are alpha defensins that are encoded by the DEFA1A3 gene found in epithelial cells of the human kidney collecting duct and produced in response to the presence of uropathogens. The DEFA1A3 gene is associated with protection from UTIs; susceptibility to UTIs increases when variations on the locus of this gene are observed. In an in vivo study, transgenic mice were created harboring the DEFA1A3 gene. The transgenic mice showed expression of HNP1–3 similar to humans. When a uropathogen was presented, namely *Escherichia coli*, the expression of the AMP was significantly amplified [66].

A study focused on the genetic polymorphisms of AMP RNase 6 showed that variations in the RNase 6 gene manifested differential bactericidal activity [66]. A single nucleotide polymorphism resulting in a substitution of ariginine for glutamine at position 66 showed significantly less bactericidal activity, as compared to the major allele with arginine. This was due to decreased lipopolysaccharide binding and bacterial agglutination, making it less likely to attach to the bacteria’s membrane and disrupt its integrity [67]. These findings demonstrate integration of pharmacogenomics to ensure greatest efficacy.

Another study focused on the defensin AMP cathelicidin LL-37 (CAMP) and its efficacy on the prevention of UTIs. CAMP, produced by the bladder’s epithelial cells and neutrophils, creates a hole in the bacterial cell’s membrane, destroying it and preventing the attachment of bacteria in the urinary tract. It also modulates inflammation and strengthens naturally occurring defenses in the urinary tract. This investigation also evaluated a link between CAMP and vitamin D, and whether vitamin D levels had any effects on the production of CAMP. Bladder epithelial cells were collected from postmenopausal women. Uropathogenic *E. coli* was introduced and a significant upregulation of CAMP was observed. It was suggested that vitamin D-induced CAMP inhibited *Escherichia coli* biofilm formation, the most common pathogen responsible for UTIs [68]. A study addressing why postmenopausal women have greater incidences of UTIs found that estrogen has the ability to amplify AMPs. Using murine models comparing ovariectomized mice to mice on estrogen replacement, as well as urothelial cell cultures with added 17β-estradiol, it was observed that estrogen not only enhanced the urinary tract epithelial by acting as a barrier against UTIs but also upregulates the secretion of the AMPs cathelicidin and β- defensins [69]. This suggests that hormone replacement therapy with estrogen in postmenopausal women could be a new approach to combatting UTIs. When the urinary tract gets invaded with uropathogenic *E. coli* and involves the kidneys, it results in a complicated form of a UTI known as pyelonephritis. A report focusing on the body’s reaction to pyelonephritis has also shown great evidence that antimicrobial peptides can help fight off infection. It was observed that the urinary tract secretes AMPs, cathelicidins, β- defensins, and RNase peptides in particular via the bladder urothelial cells, renal tubular epithelial cells, collecting duct cells, and intercalated cells. This study advocated for the kidneys to be recognized as an immune organ because of the endogenous AMPs ability to disrupt the bacteria’s membrane and help fight off infection [70]. Antimicrobial peptides, naturally produced in the body to maintain sterility, provide innate immunity and can be manipulated or amplified to intensify their effects.

A novel approach to biofilm disruption in urinary tract infections based on using an antimicrobial peptide (AMP) has been described [71]. Cecropin A (CecA) is an AMP derived from the greater wax moth *Galleria mellonella*, which, through in vivo screening, showed antibiofilm activity against uropathogenic *E. coli* (UPEC). In the study, CecA was administered together with the antibiotic nalidixic acid (NAL). Aside from any synergistic benefit, giving a patient two different antimicrobials with two completely different mechanisms of action could minimize the growing issue of antimicrobial resistance. With the two antimicrobials tested together, it was observed that the minimum inhibitory concentration (MIC) of both significantly decreased. The combination of CecA and NAL was successful in inhibiting UPEC biofilm formation. The mechanism proposed, based on electron microscopy, was the combination of CecA and NAL, which targets the adhesive filaments on UPEC, specifically the type I and P fimbriae, and limits UPEC’s ability to attach to surfaces, allowing the AMP to have contact with the bacterial membrane.

### 3.11. Quorum Sensing

QS plays a key role in biofilm formation through controlling the production of the extracellular polymer matrix, upregulating bacterial virulence genes, and facilitating resistance to antibiotics [72].

Different bacterial genes are activated at different phases of the biofilm formation process. An objective of the study by Alshammari was to evaluate the effects of knocking out genes associated with the quorum sensing process on biofilm growth and applying the approach to biofilm inhibition with urinary catheters [72]. As a model, a strain of *E. coli* was used. The genome sequence of the *E. coli* strain ATCC 25922 encodes the genes *LuxS*, *fimH*, and *bolA*. The *LuxS* gene is responsible for producing AI-2, which regulates QS and stimulates the formation of the biofilm. The *fimH* gene produces fimbriae, which is required for adhesion, and it also stimulates the formation of the biofilm. The *bolA* gene is required for curli amyloids and fimbriae production, which also contribute to the formation of the biofilm. The study design was predicated on inhibition of the growth of biofilms by disrupting these genes. CRISPR/Cas9–homology-directed repair (HDR) technology was used for gene editing. The results demonstrated that the *E. coli* wherein the genes had been knocked out was less capable of forming biofilms compared to the wildtype, with the *fimH* mutants causing the most significant decrease in biofilm formation. Interestingly, when this approach was tested on commercial urinary catheters, the results similarly showed a decrease in biofilm formation with the mutant strains.

### 3.12. Plant Bioactives

Several plants, including berry-derived botanicals; leaf- and herb-derived botanicals; and seed-, root-, and resin-derived botanicals, may provide potential therapeutic agents for UTIs due to their rich bioactive compound content, which includes phenolics, flavonoids, tannins, alkaloids, and essential oils [73]. Importantly, plant bioactive compounds exert multi-dimensional antimicrobial activities, including disruption of planktonic cell membranes, inhibition of quorum sensing and biofilm formation, and interference with bacterial metabolism [74]. These compounds also manifest anti-inflammatory, antioxidant, and immune-modulatory effects [75,76].

**Figure 5:**
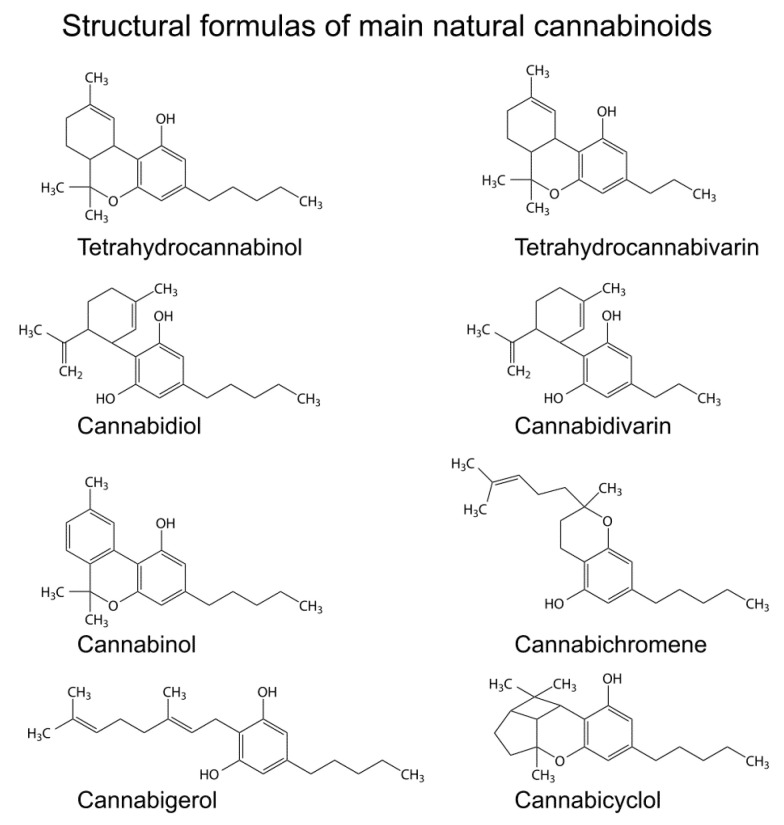
Cannabinoid structural formulas.

Recently, cannabis and its constituent components have been explored as potential therapeutic agents in some disease states [77]. The cannabinoids cannabidiol (CBD) and delta-9-tetrahydrocannabinol (THC) are the most-researched compounds found in cannabis plants (Figure 5). The effects of CBD and THC on the body are different. THC is psychoactive and affects mental processes, including mood and perception. In addition, THC manifests neuroprotective, analgesic, antiemetic, and antiglaucoma effects [78,79]. In contrast to THC, CBD is a non-psychoactive ingredient and exhibits anti-inflammatory, antioxidant, anticonvulsant, and neuroprotective effects, as well as decreasing THC psychoactivity [80,81].

Investigations have been reported recently that describe the antimicrobial potential of cannabinoids, which are found in cannabis plants. Results of numerous studies highlight the putative antimicrobial applications of multiple compounds found in cannabis plants [82]. Cannabinoids can be categorized as either endogenous or exogenous [83]. In the treatment of microbial infections, animal studies showed that exogenous cannabinoids, specifically Tetrahydrocannabinol (THC), can minimize resistance to numerous pathogens, including *Listeria monocytogenes*, *Treponema pallidum*, *Legionella pneumophila*, and *Staphylococcus aureus* [84]. Cannabidiol (CBD), another exogenous cannabinoid, has been studied in numerous in vitro studies. The in vitro studies demonstrated that CBD has both bacteriostatic and bactericidal activity against several bacteria, including methicillin-susceptible *Staphylococcus aureus* (MSSA), methicillin-resistant *Staphylococcus aureus* (MRSA), *Streptococcus mutans*, and *Streptococcus faecalis* [82]. In a recent study, CBD and cannabigerol (CBG) displayed antimicrobial effects against *Pseudomonas aeruginosa* [85]. Inhibition and eradication of *P. aeruginosa* biofilms with CBD have been demonstrated on soft contact lenses [86]. Encouraging antibacterial activity with CBD has been reported with *Salmonella typhimurium* and *Salmonella newington* [87]. The anti-biofilm activity of CBD has also been shown for the fungal pathogen Candida albicans [88]. Cannabigerol (CBG) has demonstrated anti-biofilm activity with *S. mutans*, and quorum sensing and biofilm formation of *Vibrio harveyi* were reduced in the presence of CBG [89,90]. Cannabinoids can also be useful for enhancing the activity of antibiotics when used together with antibiotics [91]. Collectively, these studies identify the diverse applications of cannabinoids as antimicrobial compounds.

Recently, integration of CBD into various drug delivery systems to enhance its therapeutic efficacy and bioavailability has been investigated. 3D-printed alginate films incorporating CBD and nanoparticles showed potential in wound-healing applications [62]. Correspondingly, 3D-printed bigel matrices and buccal films using nanostructured lipid carriers have resulted in improved cannabinoid delivery and patient compliance [92,93]. Moreover, 3D-printed stents with bioactive surfaces loaded with phytochemicals enabled sustained drug release [26,94]. In an initial study evaluating 3D-printed cannabidiol stents for UTIs, efficacy was demonstrated using the Gram-negative *Escherichia coli* and the Gram-positive methicillin-resistant *Staphylococcus aureus* (MRSA) [95]. These results are significant in that *E. coli* is highly prevalent in UTIs, and MRSA, a multidrug-resistant pathogen, illustrated the broader antimicrobial potential of the stent.

## 4. Conclusions

**Figure 6:**
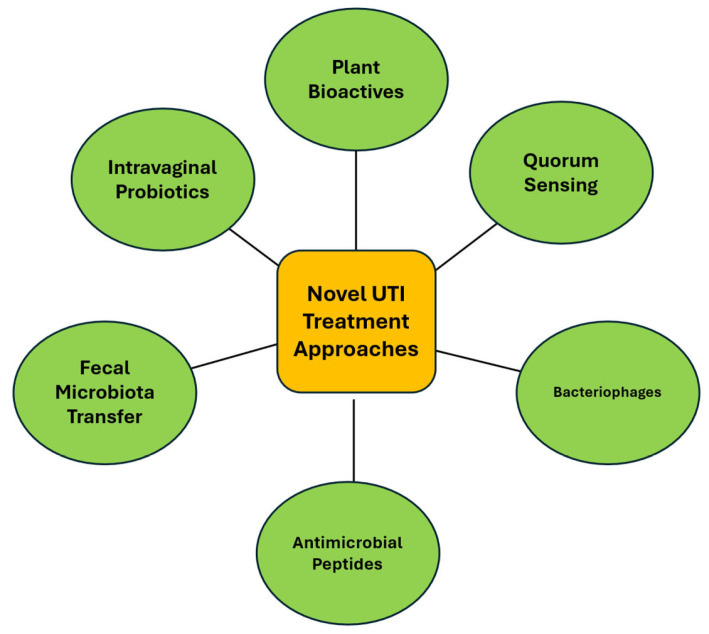
Novel approaches to urinary tract infection treatment.

Urinary tract infections are a serious public health problem. The potential for complications, including recurrent infections, negatively impacts quality of life. Currently, antibiotics are still the main therapeutic option used for the treatment of UTIs. However, the overuse of antibiotics in humans in general has been associated with the increase in antibiotic-resistant infections—a major global public health concern. Similarly, treatment of urinary tract infections has been challenging due to the increase in antimicrobial resistance. Clearly, new approaches are required to address the fundamental microbial challenge. Natural biologic products, including leveraging the healthy microbiome and the development of new synthetic entities, are showing promise (Figure 6).

Alternative approaches to antibiotic treatment for the management of recurrent infections broadly, and UTIs specifically, are warranted. Preventative strategies need to be explored. Recently, the development of bacterial vaccines has been the focus of several studies [96,97]. Vaccination against UTIs is a viable strategy. Whether vaccination will ultimately play a role as a standalone versus complementary strategy for CAUTI management remains to be determined. Given the abiotic nature of indwelling catheters, vaccination may work in combination with other methods that are designed to mitigate pathogen colonization.

Probiotics, bacteriophages, quorum sensing, antimicrobial peptides, and plant bioactives show promise as prophylactics or treatments of UTIs. These approaches, when coupled together with the administration of vaccines, suggest a potentially viable strategy for combating UTIs is possible, thereby alleviating the use of antibiotics.

## Figures and Tables

**Table 1:** Search terms used.

Urinary tract infection
UTI
Catheter
Microbiome
Biofilm
Dysbiosis
Quorum sensing
Antimicrobial peptide
Bacteriophage therapy
Probiotics
Cannabidiol
CBD

## Data Availability

No new data were created or analyzed in this study.

## References

[1] Mancuso G., Midiri A., Gerace E., Marra M., Zummo S., Biondo C. (2023). Urinary Tract Infections: The Current Scenario and Future Prospects. Pathogens.

[2] Raphael E., Argante L., Cinconze E., Nannizzi S., Belmont C., Mastrangelo C.F., Allegretti Y.H., Pellegrini M., Schmidt J. (2024). Incidence and Recurrence of Urinary Tract Infections Caused by Uropathogenic *Escherichia coli*: A Retrospective Cohort Study. Res. Rep. Urol..

[3] Turcu F.L., Vacaroiu I.A., Balcangiu-Stroescu A.E., Mitrea A.R., Miricescu D., Balan D.G., Stanigut A.M. (2025). Recurrent Urinary Tract Infections in Female Patients—A Clinical Review. J. Mind Med. Sci..

[4] Luck M.E., Martin A., Punja S., Kamar J., Zuchinali P., Edgecomb A.G., Ellis J.J. (2026). Definitions and rates of treatment failure in females with uncomplicated urinary tract infection: A systematic literature review. J. Antimicrob. Chemother..

[5] Simmering J.E., Tang F., Cavanaugh J.E., Polgreen L.A., Polgreen P.M. (2017). The Increase in Hospitalizations for Urinary Tract Infections and the Associated Costs in the United States, 1998–2011. Open Forum Infect. Dis..

[6] Amábile-Cuevas C.F. (2022). Myths and Misconceptions around Antibiotic Resistance: Time to Get Rid of Them. Infect. Chemother..

[7] Yoo J.-H. (2025). Antimicrobial Resistance—The ‘Real’ Pandemic We Are Unaware Of, Yet Nearby. J. Korean Med. Sci..

[8] Fleming-Dutra K.E., Hersh A.L., Shapiro D.J., Bartoces M., Enns E.A., File T.M., Finkelstein J.A., Gerber J.S., Hyun D.Y., Linder J.A. (2016). Prevalence of Inappropriate Antibiotic Prescriptions Among US Ambulatory Care Visits, 2010–2011. JAMA.

[9] Outpatient Antibiotic Prescriptions—United States, 2022. https://archive.cdc.gov/www_cdc_gov/antibiotic-use/data/report-2022.html?utm_source=chatgpt.com.

[10] (2024). GBD 2021 Antimicrobial Resistance Collaborators. Global burden of bacterial antimicrobial resistance 1990–2021: A systematic analysis with forecasts to 2050. Lancet.

[11] Ho C.S., Wong C.T.H., Aung T.T., Lakshminarayanan R., Mehta J.S., Rauz S., McNally A., Kintses B., Peacock S.J., de la Fuente-Nunez C. (2024). Antimicrobial resistance: A concise update. Lancet Microbe.

[12] Klein E.Y., Impalli I., Poleon S., Denoel P., Cipriano M., Van Boeckel T.P., Pecetta S., Bloom D.E., Nandi A. (2024). Global trends in antibiotic consumption during 2016–2023 and future projections through 2030. Proc. Natl. Acad. Sci. USA.

[13] Uddin T.M., Chakraborty A.J., Khusro A., Zidan B.R.M., Mitra S., Bin Emran T., Dhama K., Ripon K.H., Gajdács M., Sahibzada M.U.K. (2021). Antibiotic resistance in microbes: History, mechanisms, therapeutic strategies and future prospects. J. Infect. Public Health.

[14] Narla A.V., Borenstein D.B., Wingreen N.S. (2021). A biophysical limit for quorum sensing in biofilms. Proc. Natl. Acad. Sci. USA.

[15] Flores-Mireles A.L., Walker J.N., Caparon M., Hultgren S.J. (2015). Urinary tract infections: Epidemiology, mechanisms of infection and treatment options. Nat. Rev. Microbiol..

[16] Abu Lila A.S., Rajab A.A.H., Abdallah M.H., Rizvi S.M.D., Moin A., Khafagy E.-S., Tabrez S., Hegazy W.A.H. (2023). Biofilm Lifestyle in Recurrent Urinary Tract Infections. Life.

[17] Ramage G., Zalewska A., Cameron D.A., Sherry L., Murray C., Finnegan M.B., Loewy Z.G., Jagger D.C. (2012). A comparative in vitro study of two denture cleaning techniques as an effective strategy for inhibiting *Candida albicans* biofilms on denture surfaces and reducing inflammation. J. Prosthodont..

[18] Talapko J., Juzbašić M., Matijević T., Pustijanac E., Bekić S., Kotris I., Škrlec I. (2021). *Candida albicans*—The Virulence Factors and Clinical Manifestations of Infection. J. Fungi.

[19] Flores-Mireles A., Hreha T.N., Hunstad D.A. (2019). Pathophysiology, Treatment, and Prevention of Catheter-Associated Urinary Tract Infection. Top. Spinal Cord. Inj. Rehabil..

[20] Trautner B.W., Darouiche R.O. (2004). Role of biofilm in catheter-associated urinary tract infection. Am. J. Infect. Control..

[21] Berger I., Loewy Z.G. (2024). Antimicrobial Resistance and Novel Alternative Approaches to Conventional Antibiotics. Bacteria.

[22] Sharma D., Misba L., Khan A.U. (2019). Antibiotics versus biofilm: An emerging battleground in microbial communities. Antimicrob. Resist. Infect. Control.

[23] Pearce M.M., Hilt E.E., Rosenfeld A.B., Zilliox M.J., Thomas-White K., Fok C., Kliethermes S., Schreckenberger P.C., Brubaker L., Gai X. (2014). The Female Urinary Microbiome: A Comparison of Women with and without Urgency Urinary Incontinence. mBio.

[24] Naji A., Siskin D., Woodworth M.H., Lee J.R., Kraft C.S., Mehta N. (2024). The Role of the Gut, Urine, and Vaginal Microbiomes in the Pathogenesis of Urinary Tract Infection in Women and Consideration of Microbiome Therapeutics. Open Forum Infect. Dis..

[25] Foxman B. (2010). The epidemiology of urinary tract infection. Nat. Rev. Urol..

[26] Daniaux M., Gruber L., De Zordo T., Geiger-Gritsch S., Amort B., Santner W., Egle D., Baltzer P.A.T. (2023). Preoperative staging by multimodal imaging in newly diagnosed breast cancer: Diagnostic performance of contrast-enhanced spectral mammography compared to conventional mammography, ultrasound, and MRI. Eur. J. Radiol..

[27] Nicolle L.E. (2014). Catheter associated urinary tract infections. Antimicrob. Resist. Infect. Control..

[28] Umscheid C.A., Mitchell M.D., Doshi J.A., Agarwal R., Williams K., Brennan P.J. (2011). Estimating the Proportion of Healthcare-Associated Infections That Are Reasonably Preventable and the Related Mortality and Costs. Infect. Control. Hosp. Epidemiol..

[29] Chenoweth C.E., Gould C.V., Saint S. (2013). Diagnosis, Management, and Prevention of Catheter-Associated Urinary Tract Infections. Infect. Dis. Clin. N. Am..

[30] Stevenson K.B., Moore J., Colwell H., Sleeper B. (2005). Standardized Infection Surveillance in Long-Term Care Interfacility Comparisons From a Regional Cohort of Facilities. Infect. Control. Hosp. Epidemiol..

[31] Peng D., Li X., Liu P., Luo M., Chen S., Su K., Zhang Z., He Q., Qiu J., Li Y. (2018). Epidemiology of pathogens and antimicrobial resistanceof catheter-associated urinary tract infections in intensivecare units: A systematic review and meta-analysis. Am. J. Infect. Control..

[32] Chant C., Smith O.M., Marshall J.C., Friedrich J.O. (2011). Relationship of catheter-associated urinary tract infection to mortality and length of stay in critically ill patients: A systematic review and meta-analysis of observational studies. Crit. Care Med..

[33] Hooton T.M., Bradley S.F., Cardenas D.D., Colgan R., Geerlings S.E., Rice J.C., Saint S., Schaeffer A.J., Tambayh P.A., Tenke P. (2010). Diagnosis, Prevention, and Treatment of Catheter-Associated Urinary Tract Infection in Adults: 2009 International Clinical Practice Guidelines from the Infectious Diseases Society of America. Clin. Infect. Dis..

[34] Parsons C.L. (1986). Pathogenesis of Urinary Tract Infections. Urol. Clin. N. Am..

[35] Flores-Mireles A.L., Pinkner J.S., Caparon M.G., Hultgren S.J. (2014). EbpA vaccine antibodies block binding of *Enterococcus faecalis* to fibrinogen to prevent catheter-associated bladder infection in mice. Sci. Transl. Med..

[36] Werneburg G.T., Nguyen A., Henderson N.S., Rackley R.R., Shoskes D.A., Le Sueur A.L., Corcoran A.T., Katz A.E., Kim J., Rohan A.J. (2020). The Natural History and Composition of Urinary Catheter Biofilms: Early Uropathogen Colonization with Intraluminal and Distal Predominance. J. Urol..

[37] Hadjifrangiskou M., Kostakioti M., Chen S.L., Henderson J.P., Greene S.E., Hultgren S.J. (2011). A central metabolic circuit controlled by QseC in pathogenic *Escherichia coli*. Mol. Microbiol..

[38] Foxman B. (2014). Urinary Tract Infection Syndromes: Occurrence, recurrence, bacteriology, risk factors, and disease burden. Infect. Dis. Clin. N. Am..

[39] Danese P.N., Pratt L.A., Dove S.L., Kolter R. (2000). The outer membrane protein, Antigen 43, mediates cell-to-cell interactions within *Escherichia coli* biofilms. Mol. Microbiol..

[40] Guiton P.S., Cusumano C.K., Kline K.A., Dodson K.W., Han Z., Janetka J.W., Henderson J.P., Caparon M.G., Hultgren S.J. (2012). Combinatorial Small-Molecule Therapy Prevents Uropathogenic *Escherichia coli* Catheter-Associated Urinary Tract Infections in Mice. Antimicrob. Agents Chemother..

[41] Mittal R., Aggarwal S., Sharma S., Chhibber S., Harjai K. (2009). Urinary tract infections caused by *Pseudomonas aeruginosa*: A minireview. J. Infect. Public Health.

[42] Wang J.-W., Kuo C.-H., Kuo F.-C., Wang Y.-K., Hsu W.-H., Yu F.-J., Hu H.-M., Hsu P.-I., Wang J.-Y., Wu D.-C. (2019). Fecal microbiota transplantation: Review and update. J. Formos. Med. Assoc..

[43] Stalenhoef J.E., Terveer E.M., Knetsch C.W., Hof P.J.V., Vlasveld I.N., Keller J.J., Visser L.G., Kuijper E.J. (2017). Fecal Microbiota Transfer for Multidrug-Resistant Gram-Negatives: A Clinical Success Combined With Microbiological Failure. Open Forum Infect. Dis..

[44] Wang T., Kraft C.S., Woodworth M.H., Dhere T., Eaton M.E. (2018). Fecal Microbiota Transplant for Refractory *Clostridium difficile* Infection Interrupts 25-Year History of Recurrent Urinary Tract Infections. Open Forum Infect. Dis..

[45] Biehl L.M., Aguilar R.C., Farowski F., Hahn W., Nowag A., Wisplinghoff H., Vehreschild M.J.G.T. (2018). Fecal microbiota transplantation in a kidney transplant recipient with recurrent urinary tract infection. Infection.

[46] Hocquart M., Pham T., Kuete E., Tomei E., Lagier J.C., Raoult D. (2019). Successful Fecal Microbiota Transplantation in a Patient Suffering From Irritable Bowel Syndrome and Recurrent Urinary Tract Infections. Open Forum Infect. Dis..

[47] Ramos-Martínez A., Martínez-Ruiz R., Múñez-Rubio E., Valencia-Alijo A., Ferre-Aracil C., Vera-Mendoza M. (2020). Effect of faecal microbiota transplantation on recurrent urinary tract infection in a patient with long-term suprapubic urinary catheter. J. Hosp. Infect..

[48] Jeney S.E.S., Lane F., Oliver A., Whiteson K., Dutta S. (2020). Fecal Microbiota Transplantation for the Treatment of Refractory Recurrent Urinary Tract Infection. Obs. Gynecol..

[49] Aira A., Rubio E., Gómez A.V., Fehér C., Casals-Pascual C., González B., Morata L., Rico V., Soriano A. (2020). rUTI Resolution After FMT for *Clostridioides difficile* Infection: A Case Report. Infect. Dis. Ther..

[50] Mazumder R., Hussain A., Bhadra B., Phelan J., Campino S., Clark T.G., Mondal D. (2023). Case report: A successfully treated case of community-acquired urinary tract infection due to *Klebsiella aerogenes* in Bangladesh. Front. Med..

[51] Barrios-Villa E., Mendez-Pfeiffer P., Valencia D., Caporal-Hernandez L., Ballesteros-Monrreal M.G. (2022). Intracellular bacterial communities in patient with recurrent urinary tract infection caused by *Staphylococcus* spp and *Streptococcus agalactiae*: A case report and literature review. Afr. J. Urol..

[52] Sihra N., Goodman A., Zakri R., Sahai A., Malde S. (2018). Nonantibiotic prevention and management of recurrent urinary tract infection. Nat. Rev. Urol..

[53] Crocetto F., Balsamo R., Amicuzi U., De Luca L., Falcone A., Mirto B.F., Giampaglia G., Ferretti G., Capone F., Machiella F. (2023). Novel Key Ingredients in Urinary Tract Health—The Role of D-mannose, Chondroitin Sulphate, Hyaluronic Acid, and *N*-acetylcysteine in Urinary Tract Infections (Uroial PLUS^®^). Nutrients.

[54] Stapleton A.E., Au-Yeung M., Hooton T.M., Fredricks D.N., Roberts P.L., Czaja C.A., Yarova-Yarovaya Y., Fiedler T., Cox M., Stamm W.E. (2011). Randomized, Placebo-Controlled Phase 2 Trial of a *Lactobacillus crispatus* Probiotic Given Intravaginally for Prevention of Recurrent Urinary Tract Infection. Clin. Infect. Dis..

[55] Trautner B.W. (2010). Management of catheter-associated urinary tract infection. Curr. Opin. Infect. Dis..

[56] Mackie S.L., Koduri G., Hill C.L., Wakefield R.J., Hutchings R., Loy C., Dasgupta B., Wyatt J.C. (2015). Accuracy of musculoskeletal imaging for the diagnosis of polymyalgia rheumatica: Systematic review. RMD Open.

[57] Doolittle M.M., Cooney J.J., Caldwell D.E. (1995). Lytic infection of *Escherichia coli* biofilms by bacteriophage T4. Can. J. Microbiol..

[58] Chibeu A., Lingohr E.J., Masson L., Manges A., Harel J., Ackermann H.-W., Kropinski A.M., Boerlin P. (2012). Bacteriophages with the Ability to Degrade Uropathogenic *Escherichia coli* Biofilms. Viruses.

[59] Sanchez B.C., Heckmann E.R., Green S.I., Clark J.R., Kaplan H.B., Ramig R.F., Muldrew K.L., Hines-Munson C., Skelton F., Trautner B.W. (2022). Corrigendum: Development of phage cocktails to treat *E. coli* catheter-associated urinary tract infection and associated biofilms. Front. Microbiol..

[60] Fu W., Forster T., Mayer O., Curtin J.J., Lehman S.M., Donlan R.M. (2010). Bacteriophage Cocktail for the Prevention of Biofilm Formation by *Pseudomonas aeruginosa* on Catheters in an In Vitro Model System. Antimicrob. Agents Chemother..

[61] Letkiewicz S., Łusiak-Szelachowska M., Międzybrodzki R., Żaczek M., Weber-Dąbrowska B., Górski A. (2021). Low Immunogenicity of Intravesical Phage Therapy for Urogenitary Tract Infections. Antibiotics.

[62] Wang S.-M., Wu J.-X., Gunawan H., Tu R.-Q. (2023). Optimization of Machining Parameters for Corner Accuracy Improvement for WEDM Processing. Appl. Sci..

[63] Leitner L., Ujmajuridze A., Chanishvili N., Goderdzishvili M., Chkonia I., Rigvava S., Chkhotua A., Changashvili G., McCallin S., Schneider M.P. (2021). Intravesical bacteriophages for treating urinary tract infections in patients undergoing transurethral resection of the prostate: A randomised, placebo-controlled, double-blind clinical trial. Lancet Infect. Dis..

[64] Ali A.S., Townes C.L., Hall J., Pickard R.S. (2009). Maintaining a Sterile Urinary Tract: The Role of Antimicrobial Peptides. J. Urol..

[65] Schwartz L., Bochter M.S., Simoni A., Bender K., Rosado J.d.D.R., Cotzomi-Ortega I., Sanchez-Zamora Y.I., Becknell B., Linn S., Li B. (2023). Repurposing HDAC inhibitors to enhance ribonuclease 4 and 7 expression and reduce urinary tract infection. Proc. Natl. Acad. Sci. USA.

[66] Canas J.J., Liang D., Saxena V., Hooks J., Arregui S.W., Gao H., Liu Y., Kish D., Linn S.C., Bdeir K. (2022). Human neutrophil peptides 1-3 protect the murine urinary tract from uropathogenic *Escherichia coli* challenge. Proc. Natl. Acad. Sci. USA.

[67] Anguita R., Prats-Ejarque G., Moussaoui M., Becknell B., Boix E. (2024). A Common Polymorphism in *RNASE6* Impacts Its Antimicrobial Activity toward Uropathogenic *Escherichia coli*. Int. J. Mol. Sci..

[68] Hertting O., Holm Å., Lüthje P., Brauner H., Dyrdak R., Jonasson A.F., Wiklund P., Chromek M., Brauner A. (2010). Vitamin D Induction of the Human Antimicrobial Peptide Cathelicidin in the Urinary Bladder. PLoS ONE.

[69] Lüthje P., Hirschberg A.L., Brauner A. (2014). Estrogenic action on innate defense mechanisms in the urinary tract. Maturitas.

[70] Becknell B., Schwaderer A., Hains D.S., Spencer J.D. (2015). Amplifying renal immunity: The role of antimicrobial peptides in pyelonephritis. Nat. Rev. Nephrol..

[71] Kalsy M., Tonk M., Hardt M., Dobrindt U., Zdybicka-Barabas A., Cytrynska M., Vilcinskas A., Mukherjee K. (2020). The insect antimicrobial peptide cecropin A disrupts uropathogenic *Escherichia coli* biofilms. npj Biofilms Microbiomes.

[72] Alshammari M., Ahmad A., AlKhulaifi M., Al Farraj D., Alsudir S., Alarawi M., Takashi G., Alyamani E. (2023). Reduction of biofilm formation of *Escherichia coli* by targeting quorum sensing and adhesion genes using the CRISPR/Cas9-HDR approach, and its clinical application on urinary catheter. J. Infect. Public Health.

[73] Gadisa E., Tadesse E. (2021). Antimicrobial activity of medicinal plants used for urinary tract infections in pastoralist community in Ethiopia. BMC Complement. Med. Ther..

[74] Hemaiswarya S., Kruthiventi A.K., Doble M. (2008). Synergism between natural products and antibiotics against infectious diseases. Phytomedicine.

[75] Cipriani C., Carilli M., Rizzo M., Miele M.T., Sinibaldi-Vallebona P., Matteucci C., Bove P., Balestrieri E. (2025). Bioactive Compounds as Alternative Approaches for Preventing Urinary Tract Infections in the Era of Antibiotic Resistance. Antibiotics.

[76] Maisto M., Iannuzzo F., Novellino E., Schiano E., Piccolo V., Tenore G.C. (2023). Natural Polyphenols for Prevention and Treatment of Urinary Tract Infections. Int. J. Mol. Sci..

[77] Babayeva M., Assefa H., Basu P., Loewy Z. (2022). Autism and associated disorders: Cannabis as a potential therapy. Front. Biosci..

[78] Fishbein M., Gov S., Assaf F., Gafni M., Keren O., Sarne Y. (2012). Long-term behavioral and biochemical effects of an ultra-low dose of Δ9-tetrahydrocannabinol (THC): Neuroprotection and ERK signaling. Exp. Brain Res..

[79] Nahas G., Harvey D.J., Sutin K., Turndorf H., Cancro R. (2002). A molecular basis of the therapeutic and psychoactive properties of cannabis (Δ9-tetrahydrocannabinol). Prog. Neuro-Psychopharmacol. Biol. Psychiatry.

[80] Babayeva M., Loewy Z.G. (2023). Cannabis Pharmacogenomics: A Path to Personalized Medicine. Curr. Issues Mol. Biol..

[81] Furgiuele A., Cosentino M., Ferrari M., Marino F. (2021). Immunomodulatory Potential of Cannabidiol in Multiple Sclerosis: A Systematic Review. J. Neuroimmune Pharmacol..

[82] Barak T., Sharon E., Steinberg D., Feldman M., Sionov R.V., Shalish M. (2022). Anti-Bacterial Effect of Cannabidiol against the Cariogenic *Streptococcus mutans* Bacterium: An In Vitro Study. Int. J. Mol. Sci..

[83] Kovacs F.E., Knop T., Urbanski M.J., Freiman I., Freiman T.M., Feuerstein T.J., Zentner J., Szabo B. (2011). Exogenous and Endogenous Cannabinoids Suppress Inhibitory Neurotransmission in the Human Neocortex. Neuropsychopharmacology.

[84] Śledziński P., Nowak-Terpiłowska A., Zeyland J. (2020). Cannabinoids in Medicine: Cancer, Immunity, and Microbial Diseases. Int. J. Mol. Sci..

[85] Kagan A., Rotering S., Loewy Z.G. (2025). Anti-Biofilm Effect of Cannabinoids on a Clinical Isolate of *Pseudomonas aeruginosa*. Adv. Infect. Dis..

[86] Di Onofrio V., Gesuele R., Maione A., Liguori G., Liguori R., Guida M., Nigro R., Galdiero E. (2019). Prevention of *Pseudomonas aeruginosa* Biofilm Formation on Soft Contact Lenses by *Allium sativum* Fermented Extract (BGE) and Cannabinol Oil Extract (CBD). Antibiotics.

[87] Gildea L., Ayariga J.A., Ajayi O.S., Xu J., Villafane R., Samuel-Foo M. (2022). *Cannabis sativa* CBD Extract Shows Promising Antibacterial Activity against *Salmonella typhimurium* and *S. newington*. Molecules.

[88] Feldman M., Sionov R.V., Mechoulam R., Steinberg D. (2021). Anti-Biofilm Activity of Cannabidiol against *Candida albicans*. Microorganisms.

[89] Aqawi M., Sionov R.V., Gallily R., Friedman M., Steinberg D. (2021). Anti-Biofilm Activity of Cannabigerol against *Streptococcus mutans*. Microorganisms.

[90] Aqawi M., Gallily R., Sionov R.V., Zaks B., Friedman M., Steinberg D. (2020). Cannabigerol Prevents Quorum Sensing and Biofilm Formation of *Vibrio harveyi*. Front. Microbiol..

[91] Abichabki N., Zacharias L.V., Moreira N.C., Bellissimo-Rodrigues F., Moreira F.L., Benzi J.R.L., Ogasawara T.M.C., Ferreira J.C., Ribeiro C.M., Pavan F.R. (2022). Potential cannabidiol (CBD) repurposing as antibacterial and promising therapy of CBD plus polymyxin B (PB) against PB-resistant gram-negative bacilli. Sci. Rep..

[92] Hutchings A., Hollywood J., Lamping D.L., Pease C.T., Chakravarty K., Silverman B., Choy E.H.S., Scott D.G., Hazleman B.L., Bourke B. (2007). Clinical outcomes, quality of life, and diagnostic uncertainty in the first year of polymyalgia rheumatica. Arthritis Rheum..

[93] Gościniak A., Kocaj F., Stasiłowicz-Krzemień A., Szymański M., Karpiński T.M., Cielecka-Piontek J. (2024). 3D Printed Bigel: A Novel Delivery System for Cannabidiol-Rich Hemp Extract. Gels.

[94] Pent G.J. (2020). Over-yielding in temperate silvopastures: A meta-analysis. Agrofor. Syst..

[95] Eugster R., Santschi M., Buttitta G., Olcay B., Reymond J.-L., Aleandri S., Luciani P. (2025). 3D-Printed cannabidiol stent for local treatment of urinary tract infections. Int. J. Pharm..

[96] Xiong F., Fang X., Lu Y., Lv W. (2026). From Antibiotic Resistance to Bacterial Vaccines: A New Approach to Controlling Resistant Bacterial Infections. Infect. Drug Resist..

[97] Cardinali G., Nencini E., Gul C., Rappuoli R., Sala C., Batani G. (2026). Technologies to support vaccine development against antimicrobial-resistant bacteria. Philos. Trans. R. Soc. Lond. B Biol. Sci..

